# Anesthetic Management Using the Oxygen Reserve Index for Tracheal Resection and Tracheal End-to-End Anastomosis for a Malignant Thyroid Tumor With Tracheal Invasion

**DOI:** 10.7759/cureus.35728

**Published:** 2023-03-03

**Authors:** Rika Yajima, Yusuke Ishida, Takayuki Kobayashi, Hiroyuki Uchino

**Affiliations:** 1 Anesthesiology, Tokyo Medical University Hospital, Tokyo, JPN

**Keywords:** oxygenation, anesthetic management, thyroid cancer, tracheotomy, oxygen reserve index

## Abstract

When tracheal invasion of cancerous diseases such as thyroid cancer occurs, tracheal resection followed by end-to-end anastomosis is a treatment of choice. Anesthetic management of the patient during this procedure may pose challenges, such as maintaining ventilation during tracheal dissection, resection of the tracheal invasion, and tracheal end-to-end anastomosis. Here, we have presented a case of a woman in her 50s. Computed tomography of the head and neck displayed a 31-mm mass in the medial lobe of the thyroid gland, and irregularities in the trachea and right tracheoesophageal groove. We decided to perform total thyroidectomy followed by tracheal resection and end-to-end tracheal anastomosis, as a radical treatment. Anesthetic management was successfully performed without a decrease in the peripheral blood oxygen saturation level, due to managing oxygenation by using the oxygen reserve index (ORI^TM^) monitoring during the tracheostomy, tracheal infiltration division resection, and tracheal end-to-end suturing. This case was a unique situation requiring two intraoperative tube exchanges, but the ORI monitoring of oxygenation enabled safe anesthetic management.

## Introduction

When tracheal invasion of cancerous diseases, such as thyroid cancer, occurs, tracheal resection followed by end-to-end anastomosis is a treatment of choice [1,2]. The anesthetic management of this procedure poses a challenge regarding maintaining ventilation during tracheal dissection, resection of the tracheal invasion, and tracheal end-to-end anastomosis. The preparation of a tracheal tube as appropriate for the particular situation is necessary for ventilation maintenance, and tube positioning and tube replacement as required for manipulation of the operative field. In the case reported here, the use of oxygen reserve index (ORI^TM^; Masimo Corp., Irvine, CA) to monitor oxygenation enabled us to manage the patient’s anesthesia without the development of hypoxemia.

## Case presentation

A woman in her 50s (height, 152 cm; weight, 64 kg) consulted a nearby clinic with a chief complaint of neck discomfort, and was referred to our hospital (Tokyo Medical University Hospital) for a detailed examination. As a result of the examination, thyroid cancer with tracheal and extra-esophageal invasion was diagnosed, and we decided to perform a total thyroidectomy, right D2 lymph node dissection, combined resection of part of the esophageal muscular layer, combined resection of the right recurrent nerve, tubectomy of the trachea, and endotracheal anastomosis.

**Figure 1:**
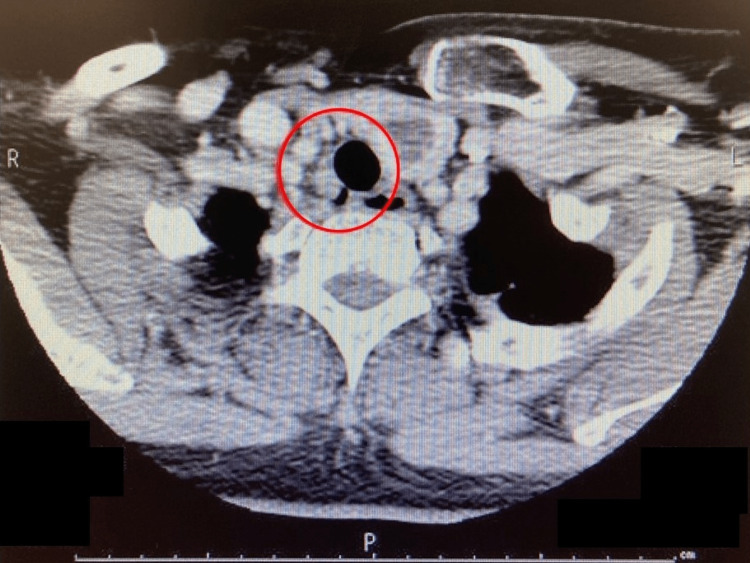
Plain chest computed tomography indicating irregularity in the right tracheoesophageal groove

**Figure 2:**
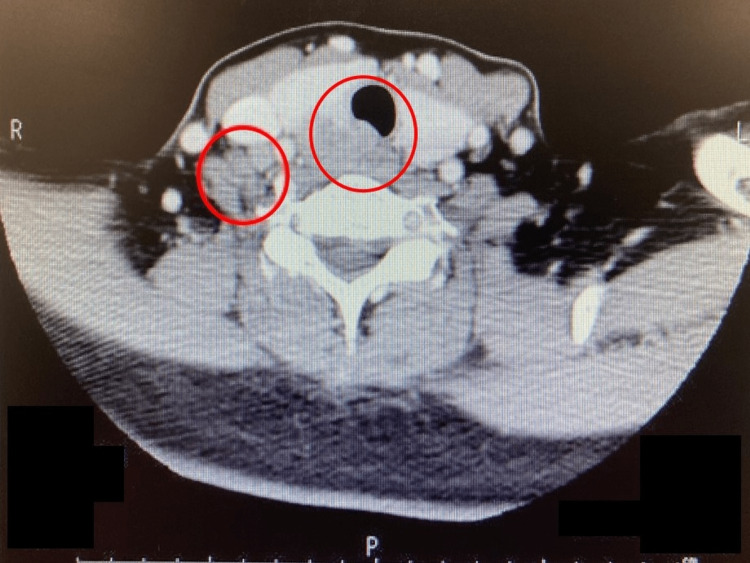
Plain chest computed tomography indicating a 31-mm mass in the medial side of the right lobe of the thyroid gland, and metastatic involvement of the right lymph node

No abnormality was observed upon the pre-operative hematological laboratory examination, and deviation of the trachea could not be identified on the thorax X-ray. Plain chest computed tomography indicated irregularity in the right tracheoesophageal groove, a 31-mm mass in the medial side of the right lobe of the thyroid gland, and metastatic involvement of the right lymph node (Figures 1, 2). Bronchoscopy displayed a slight bulge in the membranous part of the trachea, but no bilateral paralysis of the vocal cords was observed.

Anesthesia was induced using propofol at a target concentration of 4.0 μg/ml by target-controlled infusion (TCI), fentanyl (100 μg), remifentanil (0.3 μg/kg/min), and rocuronium (50 mg). Anesthesia was maintained with propofol TCI (3-4 µg/ml), remifentanil (0.1-0.2 µg/kg/min), and rocuronium (20 mg/hr). For tracheal intubation, we used a nerve integrity monitor (NIM) tube to identify the nerves, to preserve the left recurrent laryngeal nerve. ORI was monitored in addition to peripheral blood oxygen saturation (SpO_2_) as an index of intraoperative oxygenation. No significant changes in blood pressure or heart rate were observed from induction to the end of anesthesia. ORI fluctuated between 0.00 and 0.40, with a fraction of inspiratory oxygen of 0.50.

Intraoperatively, the NIM tube was removed for tracheostomy and resection of the tracheal infiltrate, and patient’s ventilation was maintained by inserting a 7.0-mm (internal diameter) laryngectomy tube into the operative field through the tracheostomy. As the patient was to temporarily be apneic during this process, changes in the ORI were carefully monitored, and no significant decrease in the SpO_2_ level occurred.

During tracheal end-to-end suturing, a surgeon inserted a tube exchanger from the tracheostomy in the operative field to the mouth, and the anesthetist used it as a guidewire for tube exchange by inserting a long spiral tube with a short cuff of 7 mm, in internal diameter, from the patient’s mouth. The ORI dropped to 0 on an occasion during a difficult tube change, but an immediate switch to ventilation from the side of the operative field prevented a significant drop in SpO_2_. By maintaining oxygenation using ORI monitoring, anesthesia could be managed in this patient without any decreases in SpO_2_. The patient was admitted to the intensive care unit under sedation and intubation, and remained sedated and intubated in the anterior cervical flexion position for the first post-operative week to prevent anastomotic overstraining and suture failure, after which she was safely extubated and returned to the general ward.

## Discussion

A drawback of common tracheotomy procedures is characterized by the occurrence of apnea duration. Anesthesia management requires paying attention to oxygenation during this period. In our case, this situation occurred twice: one at the time of tracheostomy and the other at the time of tube exchange during tracheoplasty. Therefore, we decided to use the ORI as an indicator of oxygenation management. The ORI is a monitoring device that measures the oxygenation reserve by connecting Pulse CO-Oximeter Radical-7® with the built-in rainbow SET® technology to Root® (Masimo Corp.). The ORI is a noninvasive and continuous monitoring device that displays an oxygen reserve between 0.00 and 1.00 arbitrary units in moderately hyperoxic conditions, with an arterial partial pressure of oxygen of approximately 100 to 200 mmHg [3,4]. The ORI also takes into account absorbance changes in mixed venous oxygen saturations, and thus can warn of hypoxia before a decrease in SpO_2_ occurs [5]. Szmuk et al. reported that the ORI sends a warning about 30 seconds before SpO_2_ drops to 98% [6]. Even in our case, there was a time when the procedure was continued for about 15 seconds after the ORI turned to 0, but SpO_2_ did not decrease. The ORI also dropped to 0 on an occasion during a difficult tube change, but an immediate switch to ventilation from the side of the operative field prevented a significant drop in SpO_2_. After ORI decreases and reaches 0, SpO_2_ begins to decrease; the speed to anticipate the desaturation by the ORI is much faster than SpO_2 _[7-9]. Therefore, we can plan how to handle the critical situation to prevent hypoxia.

For the maintenance of stable oxygenation, we believe that performing pure oxygenation immediately before tube exchange, and performing adequate anesthetic control, which can detect reductions in SpO_2_ by monitoring using the ORI, are important points in tracheostomy. Several reports suggest that the use of the ORI may be useful when oxygenation may deteriorate rapidly [10-12].

## Conclusions

This case was a unique situation requiring two intraoperative tube exchanges. In such cases, attention should be paid to reduced oxygenation. Even when it was difficult to replace the tube, monitoring oxygenation with the ORI enabled safe anesthesia management and avoided an emergency situation.
